# Vaccine Literacy, Covid-19 and influenza: a cross-sectional survey in Prato in the 2nd pandemic wave: Mario Bruschi

**DOI:** 10.1093/eurpub/ckac131.090

**Published:** 2022-10-25

**Authors:** M Bruschi, L Stacchini, E Stancanelli, B Velpini, V Lastrucci, F Puggelli, R Berti, C Lorini, G Bonaccorsi

**Affiliations:** 1 Department of Health Sciences, University of Florence, Florence, Italy; 2 Epidemiology Unit, Meyer Children’s University Hospital, Florence, Italy; 3 Management Department, Meyer Children’s University Hospital, Florence, Italy; 4 Department of Prevention, Azienda Sanitaria Locale Toscana Centro, Florence, Italy

## Abstract

Covid-19 is a pandemic and an infodemic, with contrasting information regarding risk and preventive measures, including vaccination. This study aims to assess Vaccine Literacy (VL) of a sample of workers in the province of Prato (Tuscany, Italy) in the second wave of the Covid-19 pandemic (November-December 2020) and to analyze the relationship between VL and attitudes about Covid-19 and flu vaccination. A cross-sectional design was adopted. Sociodemographic characteristics, health information, vaccination behaviour for past and current flu season and intention to get vaccinated against Covid-19 were collected. A multivariate logistic regression was performed to identify predictors of getting a Covid-19/flu vaccination. The Italian Health Literacy tool on Vaccination (HLVa-IT) tool was used to measure VL. A total of 117 questionnaires were analyzed. Among them, 64.9% intended to get Covid-19 vaccine. The mean VL was 3.18 ± 0.43 (functional 2.87 ± 0.72; interactive-critical 3.36 ± 0.45) out of 4. Having more than one comorbidity was a negative predictor of intention to get Covid-19 vaccine (OR: 0.21 95%CI: 0.04 - 0.91). Regarding the flu vaccine, being vaccinated in the previous season was the only positive predictor of being vaccinated in the current season (OR = 24.25 95%CI 7.96 - 87.73). The study was conducted before the authorization of Covid-19 vaccines: little information about them may have contributed to VL not being related to the intention to get vaccination. The negative role of comorbidities could be due to fear of adverse effects on fragile health status. For flu vaccination, VL may have exerted a lower impact because of the positive experience with the flu vaccine in terms of safety and effectiveness in the previous seasons.

**Key messages:**

• The introduction of new vaccines should be supported by effective communication.

• Better knowledge of current vaccines and not just routine administration is desirable for greater personal empowerment.

